# Supplementary figures and images for: HSP70 via HIF-1 α SUMOylation inhibits ferroptosis inducing lung cancer recurrence after insufficient radiofrequency ablation
Source: PLoS One. 2023 Nov 10;18(11):e0294263. doi: 10.1371/journal.pone.0294263 (PMC10637661; doi:10.1371/journal.pone.0294263)

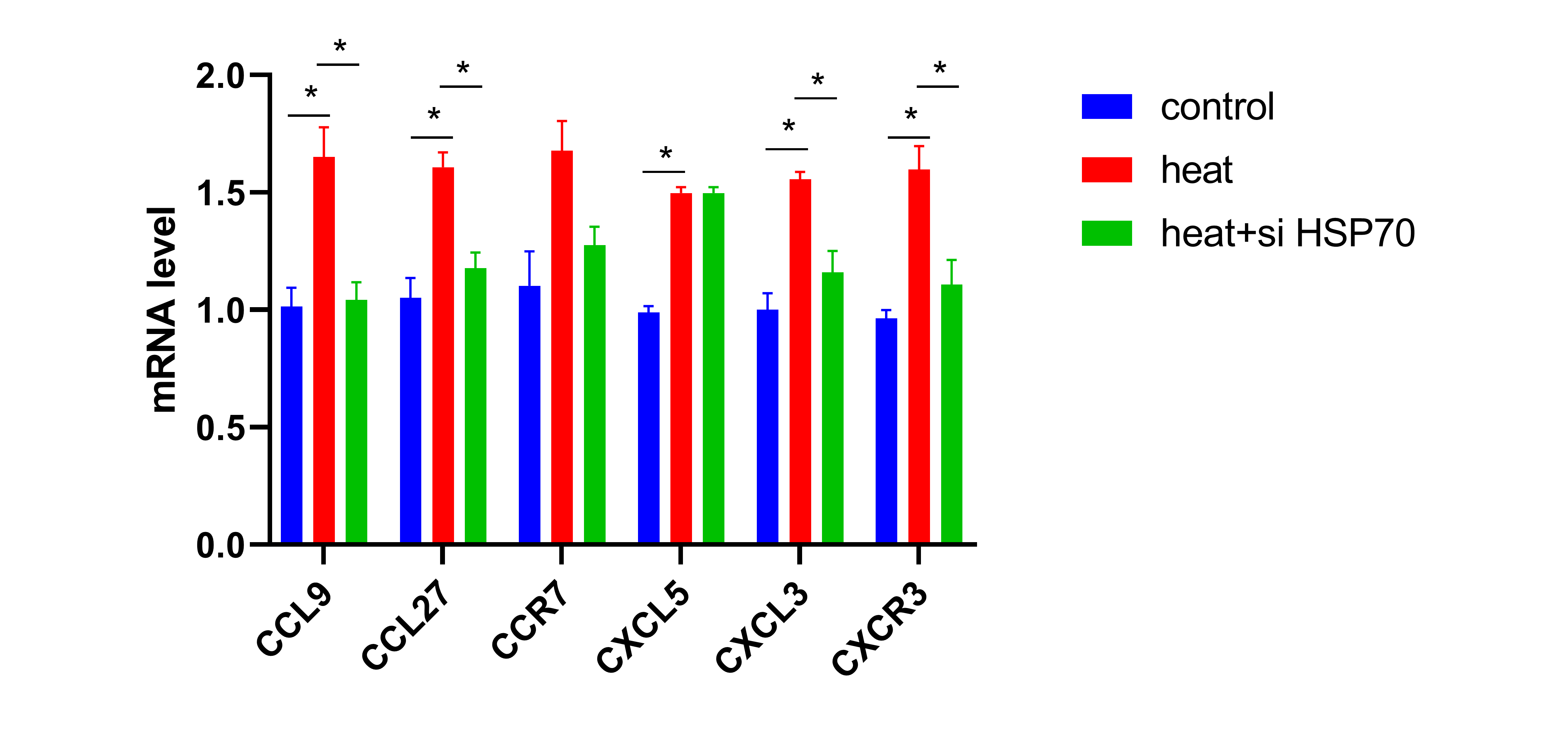

Supplement: S1 Fig — (TIF) [file pone.0294263.s001.tif]
