# Supplementary material for: HSP70 via HIF-1 α SUMOylation inhibits ferroptosis inducing lung cancer recurrence after insufficient radiofrequency ablation
Source: PLoS One. 2023 Nov 10;18(11):e0294263. doi: 10.1371/journal.pone.0294263 (PMC10637661; doi:10.1371/journal.pone.0294263)
Supplement: S1 Table — (DOCX) [file pone.0294263.s002.docx]

Supplementary Table 1: Antibody information

| **Antibody** | **Species** | **Dilution** | **Company** | **catalog** |
| --- | --- | --- | --- | --- |
| GAPDH | Rabbit | 1:10000 | abcam | ab181602 |
| SLC7A11 | Rabbit | 1:2000 | abclonal | A13685 |
| ACSL3 | Rabbit | 1:1000 | abclonal | A11679 |
| HIF-1α | Rabbit | 1:1000 | CST | #36169 |
| HSP70 | Rabbit | 1:1000 | abcam | ab181606 |
| SENP1 | Rabbit | 1:1000 | proteintech | 25349-1-AP |
| Ubc9 | Rabbit | 1:2000 | abcam | ab33044 |
| RanBP2 | Rabbit | 1:300 | abcam | ab245563 |
