# Supplementary material for: HSP70 via HIF-1 α SUMOylation inhibits ferroptosis inducing lung cancer recurrence after insufficient radiofrequency ablation
Source: PLoS One. 2023 Nov 10;18(11):e0294263. doi: 10.1371/journal.pone.0294263 (PMC10637661; doi:10.1371/journal.pone.0294263)
Supplement: S2 Table — (DOCX) [file pone.0294263.s003.docx]

Supplementary Table 2: Primer information

| Gene | Forward primer | reverse primer |
| --- | --- | --- |
| GAPDH | CATCATCCCTGCCTCTACTGG | GTGGGTGTCGCTGTTGAAGTC |
| CCL21 | TCCCAGCTATCCTGTTCTTGC | CTGTGACCGCTCAGTCCTCTT |
| CCL9 | ATTGAAATGTTTCACATGGGCT | TTGTAGGTCCGTGGTTGTGAG |
| CCR7 | CTGTTACCTTGTCATCATCCGC | GAGATCGTTGCGGAACTTGAC |
| CXCL5 | GCTGTGTTGAGAGAGCTGCG | CAGTTTTCCTTGTTTCCACCG |
| CXCL3 | CGTCCGTGGTCACTGAACTG | GTCAGTTGGTGCTCCCCTTG |
| CXCR2 | CCCAGGTCAGAAGTTTCATCGT | GGGCTTTTCACCTGTAGGACAC |
| HSP70 | GTTTCGGTTCCTTGTTTCTATACTG | TTTCTACCTCCCAATGTCGTGT |
| HIF1α | CCGATGGAAGCACTAGACAAAGT | TTTGAGGACTTGCGCTTTCAG |
| SLC7A11 | GGCAGTTGCTGGGCTGATTTA | GATGACGAAGCCAATCCCTGT |
| GPX4 | CCAGTGAGGCAAGACCGAAGT | TCCTGCTTCCCGAACTGGTTA |
| TFRC | AGAGGTCGCTGGTCAGTTCGT | CTGGAAGTAGCACGGAAGAAGTC |
| ACSL4 | GGCACAACAGAAAGGGGTAGA | ACCAAACCAGTTTCAGGGGT |
| ACSL5 | GTAGGAGTGGTGGTTCCTGAC | TGGCTTTGACTTGGTTTTGGC |
| CP | CACAGCAAACCTCTTCCCTC | AGTATGTCCTCTCTCCCAGGT |
| MAP1LC3B2 | AGCGTCTCCACACCAATCTC | TTTCATCCCGAACGTCTCCTG |
| SLC40A1 | ACTGTCCTGGGCTTTGACTG | GACCTGTCCGAACCAAACCA |
| TF | TCTCTAAGCGTTACACATGCC | CCAGTCCTTCCTCACTCCCTT |
| SAT1 | GGTTGCAATGAGGTGTCGCT | ACTGGACAGATCAGAAGCACC |
| MAP1LC3A | CATCGCGGACATCTACGAGC | GTAGAGGCAGCTCAGTTCAGG |
| GBP1 | ACATACTCCCTGAAGCTGAAGAA | CTTGTTGCACAAATTCGGGGT |
