# Supplementary material for: HSP70 via HIF-1 α SUMOylation inhibits ferroptosis inducing lung cancer recurrence after insufficient radiofrequency ablation
Source: PLoS One. 2023 Nov 10;18(11):e0294263. doi: 10.1371/journal.pone.0294263 (PMC10637661; doi:10.1371/journal.pone.0294263)

Original blot or gel images

Fig. 1B and the corresponding originals for Fig. 1B.

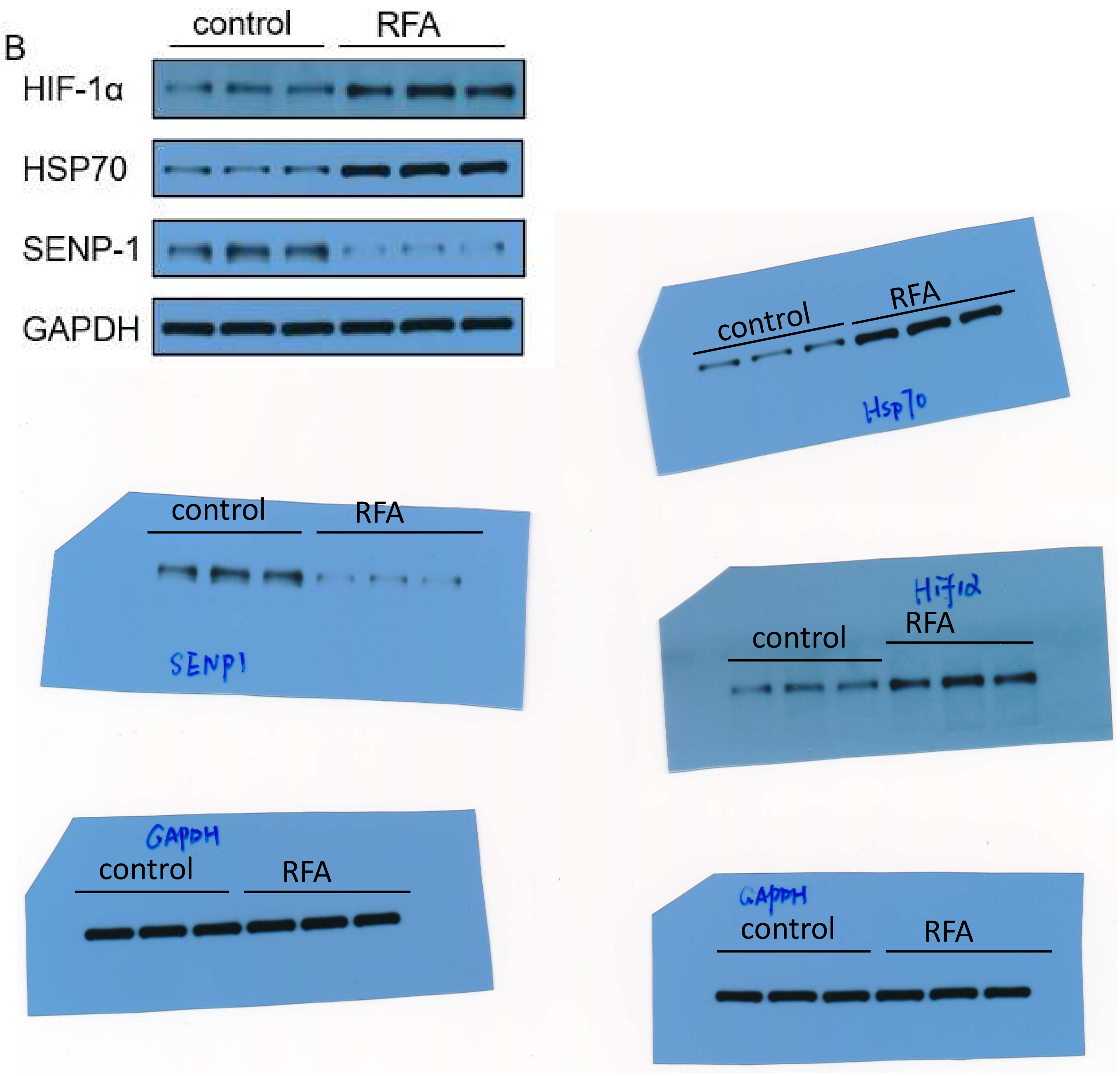

Fig. 1C and the corresponding originals for Fig. 1C.

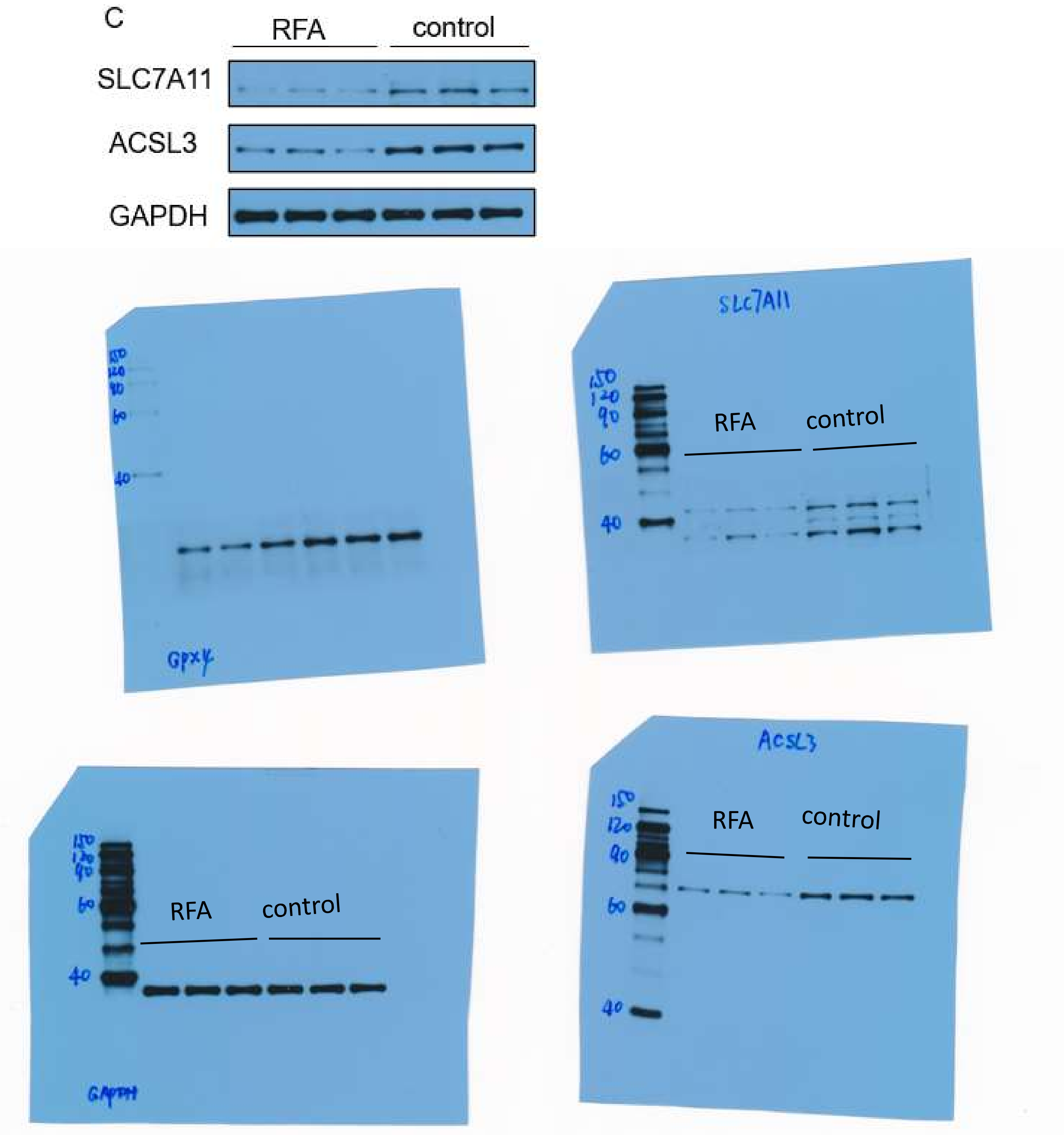

Fig. 2A and the corresponding originals for Fig. 2A.

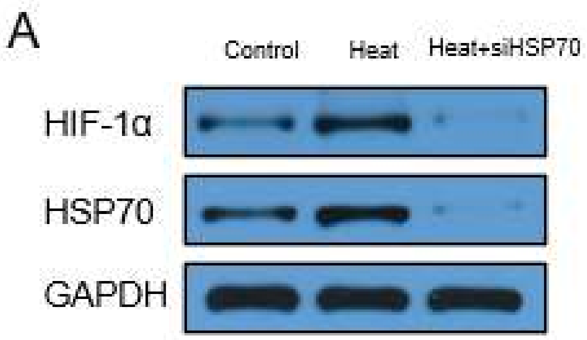

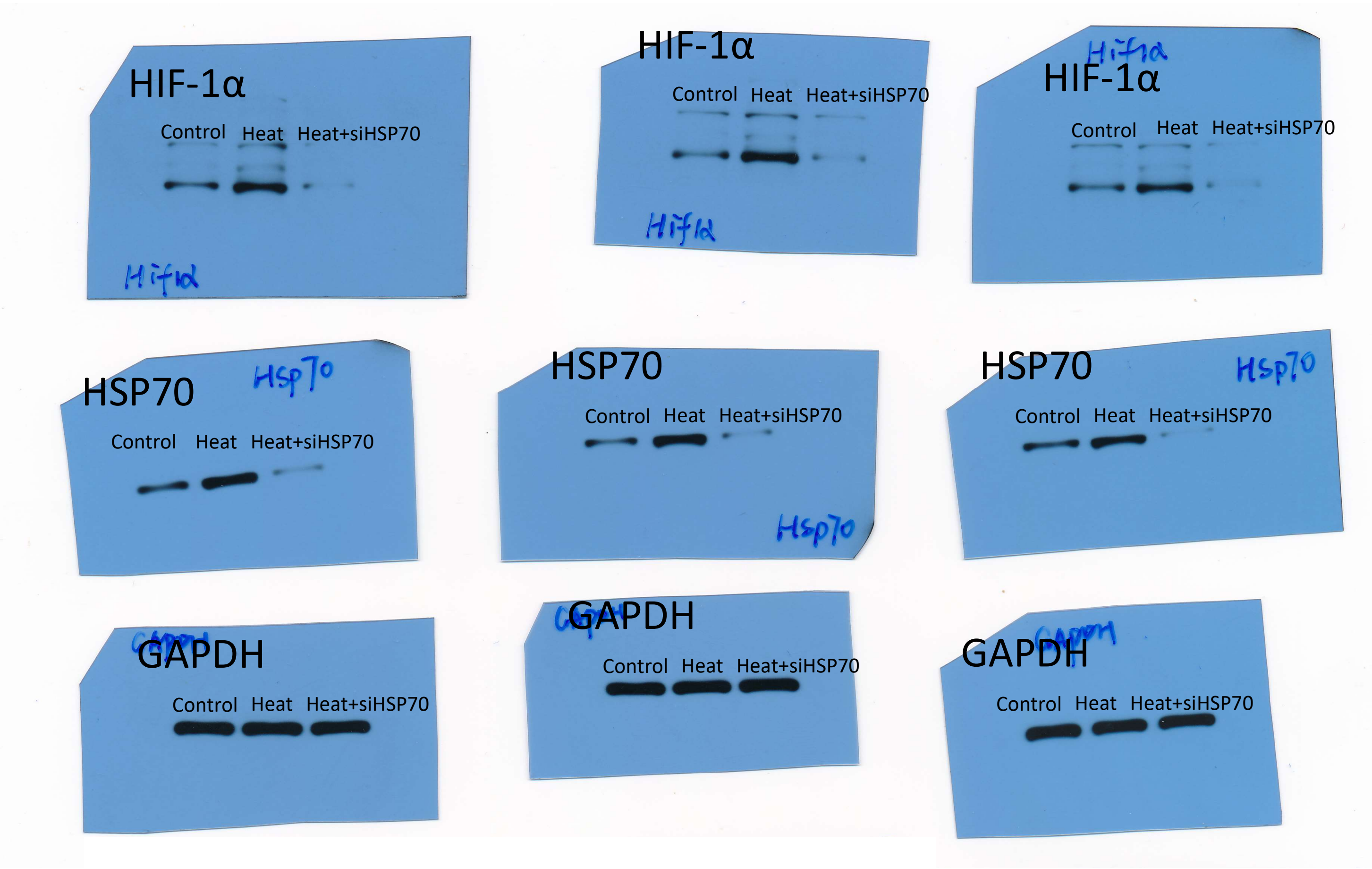

Fig. 2E and the corresponding originals for Fig. 2E.

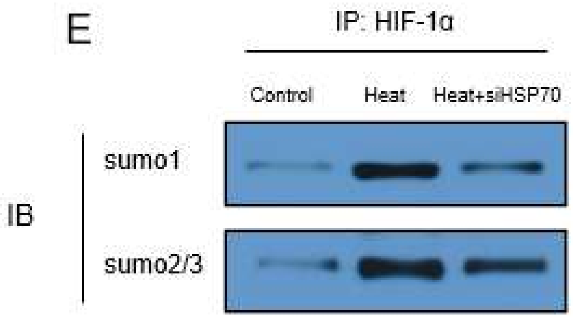

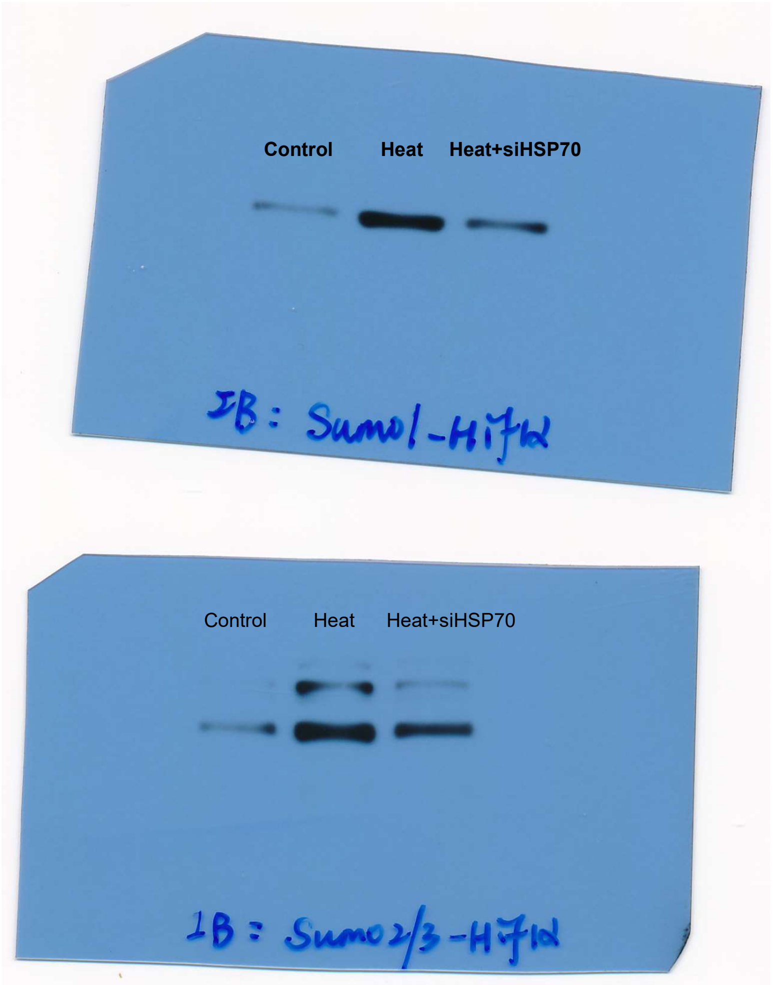

Fig. 2F and the corresponding originals for Fig. 2F.

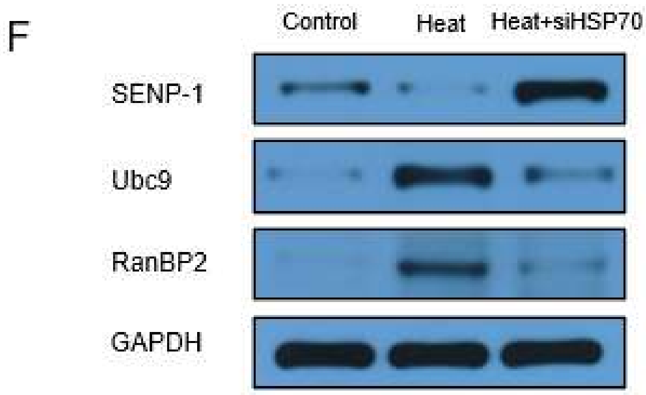

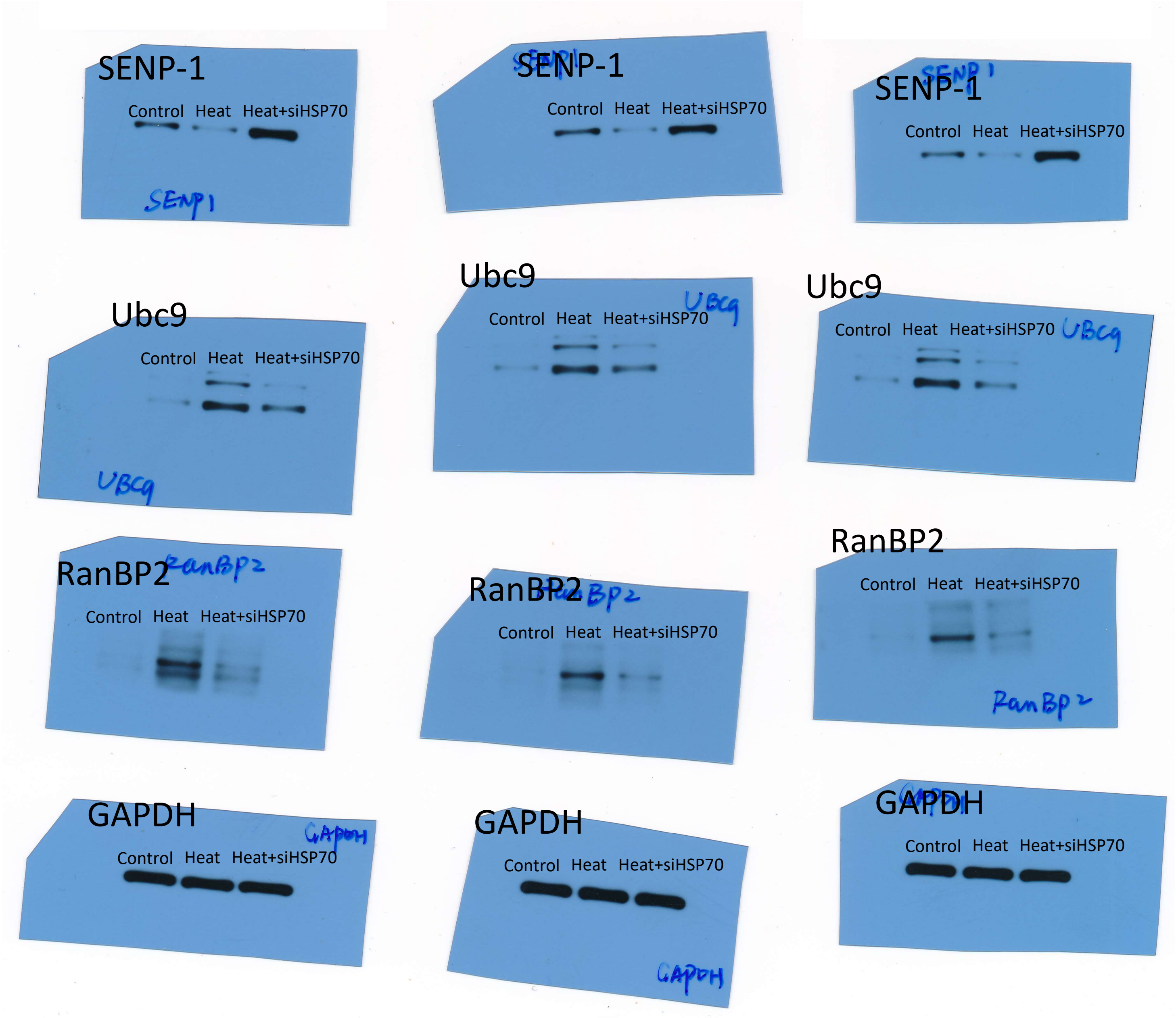

Fig. 3B and the corresponding originals for Fig. 3B.

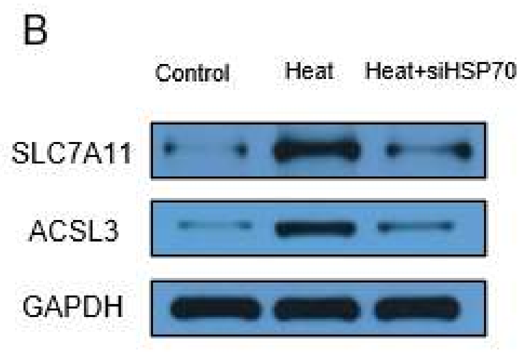

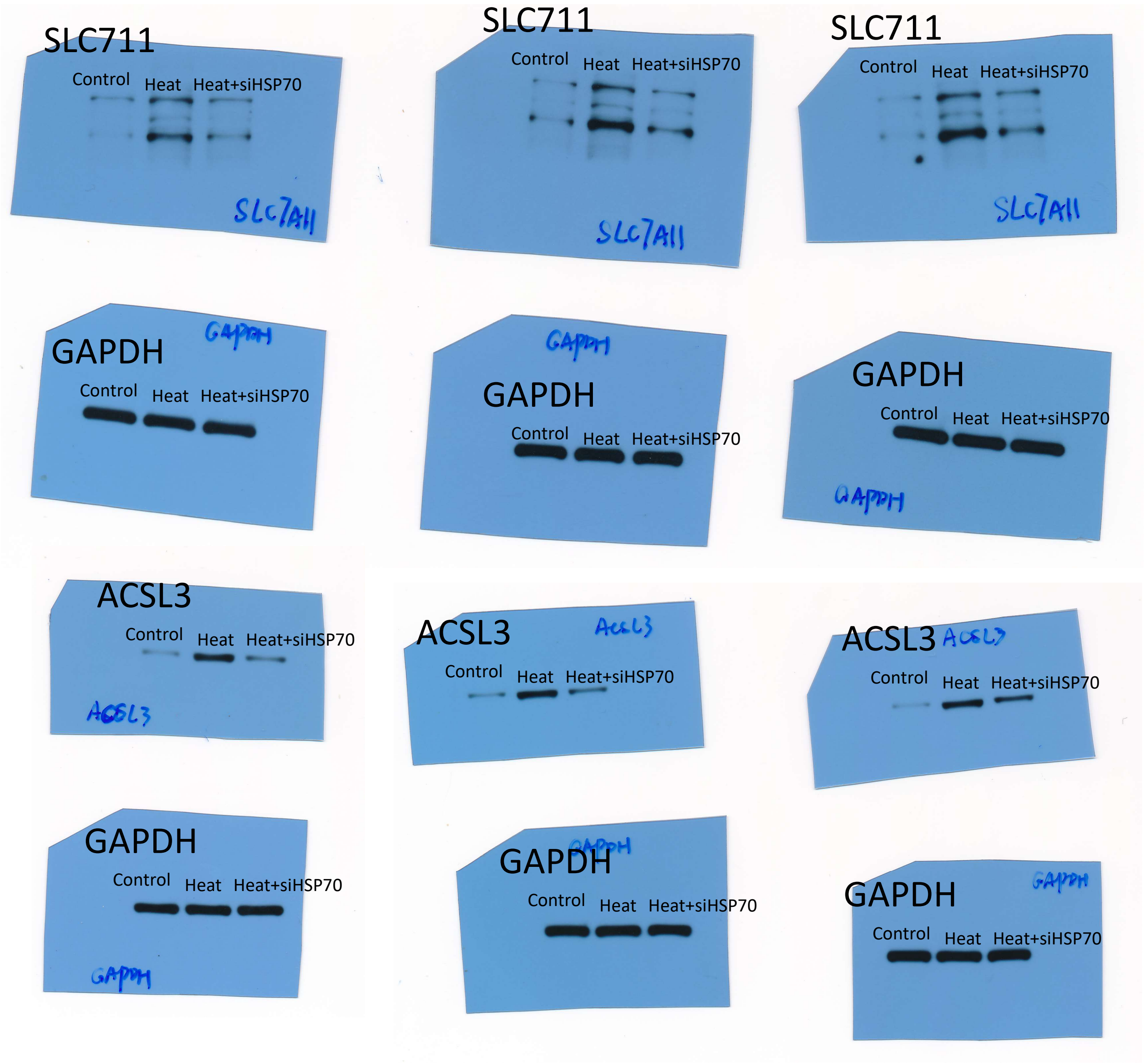

Supplement: S1 File — (PDF) [file pone.0294263.s004.pdf]
