# Supplementary material for: HSP70 via HIF-1 α SUMOylation inhibits ferroptosis inducing lung cancer recurrence after insufficient radiofrequency ablation
Source: PLoS One. 2023 Nov 10;18(11):e0294263. doi: 10.1371/journal.pone.0294263 (PMC10637661; doi:10.1371/journal.pone.0294263)
Supplement: Attachment — Submitted filename: 1 Response to Reviewers.docx [file pone.0294263.s005.docx]

PONE-D-23-06260

HSP70 via HIF-1 α SUMOylation inhibits ferroptosis inducing lung cancer recurrence after insufficient radiofrequency ablation

PLOS ONE

Dear Dr. Ruhul Amin,

We greatly appreciate the editor's comments and feedback. We have reviewed the comments and revised the manuscript, " HSP70 via HIF-1α SUMOylation inhibits ferroptosis inducing lung cancer recurrence after insufficient radiofrequency ablation" (Submission ID: PONE-D-23-06260). The following is our response to the editorial comments.

Question and response:

1. Please ensure that your manuscript meets PLOS ONE's style requirements, including those for file naming.

**Response:** We have revised the manuscript to fit the style of PLOS ONE.

2. To comply with PLOS ONE submissions requirements, in your Methods section, please provide additional information regarding the experiments involving animals and ensure you have included details on (1) methods of sacrifice, (2) methods of anesthesia and/or analgesia, and (3) efforts to alleviate suffering.

**Response:** Information needed for editor is listed in Materials and Methods-Animals and Cells, Surgical procedures and RFA treatment.

3. We noticed you have some minor occurrence of overlapping text with the following previous publication(s), which needs to be addressed:

**Response:** We have compared the manuscript with several papers presented by the editors, and have corrected those with a high degree of repetition.

4. Please provide an amended statement that declares *all* the funding or sources of support (whether external or internal to your organization) received during this study, as detailed online in our guide for authors at http://journals.plos.org/plosone/s/submit-now. Please also include the statement “There was no additional external funding received for this study.” in your updated Funding Statement.

Please include your amended Funding Statement within your cover letter. We will change the online submission form on your behalf.

**Response:** We added this to the Funding and cover letter.

5. We note that you have indicated that data from this study are available upon request. PLOS only allows data to be available upon request if there are legal or ethical restrictions on sharing data publicly. For more information on unacceptable data access restrictions, please see http://journals.plos.org/plosone/s/data-availability#loc-unacceptable-data-access-restrictions.

**Response:** The original data set generated and/or analyzed during the current study can be obtained in the SRA repository, login number [PRJNA943582], the link is http://www.ncbi.nlm.nih.gov/bioproject/943582.

6. PLOS requires an ORCID iD for the corresponding author in Editorial Manager on papers submitted after December 6th, 2016. Please ensure that you have an ORCID iD and that it is validated in Editorial Manager.

**Response:** We have already set it up.

7. Your ethics statement should only appear in the Methods section of your manuscript.

**Response:** We have modified it.

8. PLOS ONE now requires that authors provide the original uncropped and unadjusted images underlying all blot or gel results reported in a submission’s figures or Supporting Information files.

**Response:** Our original uncropped and unadjusted blot or gel image data are placed in the Supporting information file.

9. Please include captions for your Supporting Information files at the end of your manuscript, and update any in-text citations to match accordingly.

**Response:** We have added the title of the supporting information document at the end of the manuscript.

10. Thank you for stating the following in the Competing Interests section:

“The present study was supported by the Special Fundamental Research Project of Shenzhen Science and Technology Plan (Natural Science Foundation) No. JCYJ20190807144201675.”

We note that one or more of the authors have an affiliation to the commercial funders of this research study: Natural Science Foundation

**Response:** We have already made a statement about this in the **Funding** section.

Reviewers' comments:

Reviewer #1:

1. The results of this study are uncertain, because all data are based mainly on only single type cell (A549 cells). The authors need to conduct similar experiments using another type of NSCLC cell line.

**Response:** Many thanks to reviewers for your suggestions! It has been reported in the literature that after radiofrequency ablation, patients have elevated levels of serum HSP70, a good biomarker for lung cancer [1]. Our previous study on human NSCLC NCI-H1650 cell line found that HSP70/HIF-1α induced accelerated proliferation and angiogenic potential in residual lung cancer after RFA treatment, and its expression was regulated by PI3K/Akt signaling pathway [2]. Studies on A549-H, CCL-185-H and H358-H cells found that insufficient RFA could promote NSCLC growth in vitro and in vivo by upregulating HIF-1α via PI3K / Akt signaling [3]. And our present study using the A549 cell line similarly found the regulatory role of the HIF-1α/HSP70 pathway on the recurrence of lung cancer after incomplete radiofrequency ablation.

We believe that these studies have demonstrated the regulatory mechanism of the HIF-1α/HSP70 pathway in the recurrence of lung cancer in different cell types after incomplete radiofrequency ablation.

Nevertheless, we are again grateful for the suggestions made by the reviewers, which will remind us to place more value on the rigor of the protocol and logical completeness in our future studies.

Reference:

[1] Haen SP, Gouttefangeas C, Schmidt D, et al. Elevated serum levels of heat shock protein 70 can be detected after radiofrequency ablation. Cell Stress Chaperones. 2011;16(5):495-504. doi:10.1007/s12192-011-0261-y

[2] Wan J, Wu W, Huang Y, Ge W, Liu S. Incomplete radiofrequency ablation accelerates proliferation and angiogenesis of residual lung carcinomas via HSP70/HIF-1α. Oncol Rep. 2016;36(2):659-668. doi:10.3892/or.2016.4858

[3] Wan J, Wu W, Chen Y, Kang N, Zhang R. Insufficient radiofrequency ablation promotes the growth of non-small cell lung cancer cells through PI3K/Akt/HIF-1α signals. Acta Biochim Biophys Sin (Shanghai). 2016;48(4):371-377. doi:10.1093/abbs/gmw005

2. It is also difficult to obtain conclusion based on current data, because results are basically obtained from HSP70 knockdown experiments. Whether overexpression of HSP70 will demonstrate opposite results is unclear.

**Response:** Thanks for your valuable comments. In the cellular experimental design, we detected the expression of HSP70 in different treatments (Control, Heat, Heat+siHSP70) by Western blot assay (Figure 2A, figure below). The results showed that HSP70 was significantly up-regulated in the **Heat** treatment group compared to the control group, suggesting that this treatment had already indicated the overexpression treatment of HSP70. Therefore, we did not conduct further experiments on overexpression of HSP70 separately. We believe that the results of the study have demonstrated the role of HSP70.

3. Abstract: the names of SUMU-associated and ferroptosis-related markers should be mentioned.

**Response:** Thank you for your suggestions, which we have supplemented SUMO-associated and ferroptosis-related markers in the **Abstract** section

4. Materials and Methods: Statistical methods should be mentioned

**Response:** Thanks for pointing this out, we have added statistical methods in the Materials & Methods section!

5. Discussion: in the second paragraph, “posttranslational modification”, should be “posttranscriptional regulation”

**Response:** We have made the changes.

6. Figure legends: It would be better to give each Fig. a title. For example: Fig.1 Heat stress up-regulated HSP70 and HIF-1α expression but inhibited the expression of SENP-1, SCL7A11, and ACSL3…..

**Response:** Thank you for your suggestions. We have updated the figure legends of all the figures.

The above are our responses to editorial and reviewer comments. We look forward to receiving your further decisions.

Kind regards,

Jun Wan
